# Nanodelivery of Bioactive Natural Products: A Targeted Therapeutic Breakthrough for Atherosclerosis

**DOI:** 10.3390/pharmaceutics17091102

**Published:** 2025-08-25

**Authors:** Chen Liu, Peichen Wang, Renjun Gu, Keyan Zhao, Yang Gao, Bihua Tang, Mingfei Shi, Ziyun Li

**Affiliations:** 1 School of Acupuncture-Moxibustion and Tuina, School of Health Preservation and Rehabilitation, Nanjing University of Chinese Medicine, Nanjing 210023, China; liuchen@njucm.edu.cn (C.L.); 055521140@njucm.edu.cn (P.W.); 202410495@njucm.edu.cn (K.Z.); 055521123@njucm.edu.cn (Y.G.); 2 School of Chinese Medicine, Nanjing University of Chinese Medicine, Nanjing 210023, China; renjungu@njucm.edu.cn; 3 The Third Clinical Medical College, Nanjing University of Chinese Medicine, Nanjing 210023, China; 202430171@njucm.edu.cn; 4 School of Integrative Medicine, Nanjing University of Chinese Medicine, Nanjing 210023, China

**Keywords:** atherosclerosis, bioactive natural products, nanoparticle drug delivery system, targeted therapy, bioavailability

## Abstract

Atherosclerosis (AS), as a major pathogenic factor of cardiovascular diseases, remains a global health challenge due to its multifactorial nature and recalcitrant therapeutic limitations. The inherent multitarget activity of bioactive natural products (BNPs) positions them as ideal complements to conventional therapeutics. While effective in symptom management, BNPs often falter due to two critical drawbacks: insufficient targeting and poor bioavailability. Recent nanoparticle drug delivery systems (NDDSs) offer a transformative solution. This article systematically reviews the research progress on the combination of BNPs such as phenols, terpenes, and alkaloids with NDDS for the treatment of AS. By optimizing pharmacokinetic properties and targeting efficiency, NDDSs effectively address the clinical limitations of BNPs in AS treatment, including low bioavailability and poor solubility. The study analyzes various NDDS design strategies and their mechanisms in intervening AS pathological processes, such as improving drug stability, enhancing targeting, and controlled release. Additionally, it explores natural compounds with potential antioxidant, anti-inflammatory, cell transformation-regulating, and lipid metabolism-modulating effects, offering innovative approaches for AS clinical therapy.

## 1. Introduction

Atherosclerosis (AS) is the leading cause of cardiovascular diseases (CVDs) worldwide [1]. According to the World Health Organization, CVD in 2019 claimed 17.9 million lives, which is equivalent to 32% of total deaths (https://www.who.int/en/news-room/fact-sheets/detail/cardiovascular-diseases-(cvds) (accessed on 26 June 2025)). AS is a widely distributed chronic inflammatory disease of the arterial wall, characterized by lesions in the intima-media layer and plaque accumulation. Its progression, including erosion or rupture of the plaque, can lead to thrombosis and result in fatal cardiovascular events such as myocardial infarction or ischemic stroke [2,3]. In addition, the damage caused by AS to the cardiovascular and cerebrovascular systems is not limited to a local area. Instead, it can affect multiple organs throughout the body [4], and there is a trend of AS occurring at a younger age [5]. This undoubtedly poses a huge challenge to the public health and medical systems. Currently, statins are the primary drugs used to treat AS. However, all statins are rapidly absorbed upon oral administration and undergo extensive hepatic first-pass metabolism, leading to low bioavailability and complications such as rhabdomyolysis [6]. This suggests that while they improve cardiovascular health, their lack of targeting at the disease site may result in dose-dependent adverse effects. Consequently, there is a need for the development and utilization of novel drugs to address these limitations.

As an important source of new drug molecules with a wide range of biological effects and low toxicity, they have become a hotspot for a wide range of researchers to study various diseases and develop therapeutic drugs [7]. Many natural drugs such as *Salvia miltiorrhiza*, *Curcuma longa* and *Panax notoginseng* have shown ability to treat AS. Therefore, the development of highly effective and low-toxicity natural drugs is important to improve the prognosis of AS patients. BNPs derived from plants are the main source of active pharmaceutical ingredients in drugs, but most of the active ingredients in traditional Chinese medicines (TCMs), such as saponins, alkaloids, flavonoids, and volatile oils, suffer from deficiencies such as low bioavailability and poor solubility, which constrain the application of TCM in clinical applications [8,9].

Combining bioactive natural products (BNPs) with nanotechnology to form a nanparticle drug delivery system (NDDS) offers a promising approach to optimize the pharmacokinetic properties of BNPs and enhance their targeting, thereby improving the efficiency of intervention in the pathological process of AS. As an emerging drug delivery technology, NDDS demonstrates potential in overcoming traditional drug delivery challenges. By improving solubility, enhancing targeting effects, and controlling drug release, NDDS can address key limitations of conventional treatments, such as poor bioavailability and insufficient targeting at the disease site. Specifically, NDDS can (1) improve the bioavailability of poorly soluble active ingredients in TCM through physical encapsulation or chemical bonding; (2) enhance the accumulation of drugs in diseased tissues or sites through passive or active targeting mechanisms, thereby reducing systemic drug toxicity; and (3) control or maintain drug release to reduce the frequency of drug administration, improve patient medication compliance, and mitigate adverse reactions associated with fluctuations in blood drug concentration [10].

Based on these advantages, this paper focuses on the application potential of NDDS in AS treatment. We conducted a comprehensive literature search in PubMed database using the search strategy combined keywords related to the research topic “nanoparticles”, “atherosclerosis”, and “natural products”. The inclusion criteria were original studies published between 2015 and 2025 that met the following conditions: (1) being original research; (2) employing nanoparticle-encapsulated BNPs; (3) being evaluated in atherosclerosis models; and (4) reporting at least one pharmacodynamic outcome.

## 2. Application of BNPs of NDDS in AS Therapy

### 2.1. Phenols and Their Derivatives

#### 2.1.1. Curcumin (Cur)

Cur is a polyphenol compound extracted mainly from the rhizomes of *Curcuma longa*, *Curcuma zedoaria*, and *Acorus calamus* L., which possesses a variety of biological activities such as antioxidant, anti-inflammatory, and anticancer activities [11,12]. Cur can be used directly for the treatment of AS, and its mechanism of action includes modulation of gene networks and inhibition of leukocyte adhesion and transendothelial migration through NF-κB-dependent pathways [13]. However, poor water solubility and low stability of Cur [14] limit the effectiveness of its clinical application.

**Figure 1:**
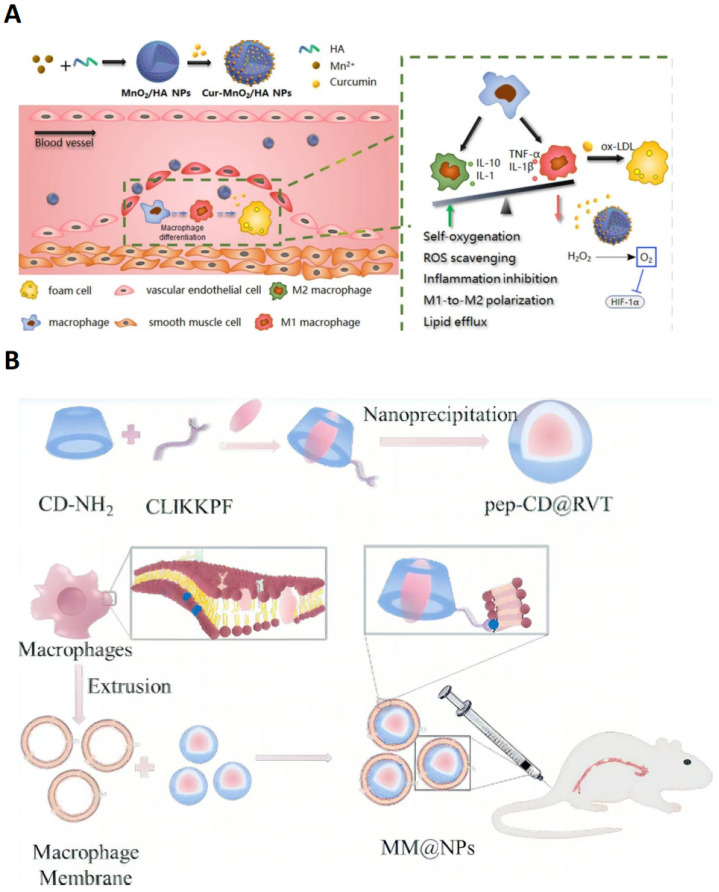
(**A**) An illustration of the preparation of the Cur-loaded MnO_2_/HA for targeting delivery in atherosclerotic lesions, and the mechanisms for anti-AS therapy. Reproduced with permission [16]. Copyright 2022, *Journal of Nanobiotechnology*. (**B**) Illustration of pep-CD@RVT spontaneously encapsulated by macrophage membranes for enhancing target therapy in atherosclerosis. Reproduced with permission [17]. Copyright 2024, American Chemical Society.

To address these challenges, several Cur-based NDDS have demonstrated the effect in AS treatment, enhancing both the utilization of Cur and its targeting efficiency through various nano-delivery techniques. For example, two NDDS, HASF@Cur and Cur-MnO_2_/HA, specifically bind to the CD44 receptor on the macrophage surface through both oligomeric hyaluronic acid (oHA) and hyaluronic acid (HA), respectively, to achieve AS targeting. The nanomicellar drug delivery system encapsulating Cur, denoted as HASF@Cur, is composed of oHA, TKL (thiolated keratin-like polymer), and Fc (ferrocene) materials. Through dual Reactive Oxygen Species (ROS)-sensitive and CD44 receptor targeting, it improves the stability and delivery efficiency of Cur. This NDDS consists of oHA as a targeting ligand, which specifically binds to the CD44 receptor on the surface of macrophages to achieve active targeting. Meanwhile, thioketal linkages and Fc-wrapped Cur form stable micelles through hydrophobic interactions and act as reactive oxygen species (ROS)-sensitive elements, which are activated (H_2_O_2_ concentration-dependent) in the high-ROS AS plaque microenvironment, triggering rapid drug release. In vitro experiments show that this design not only enhances the aqueous solubility and bioavailability of Cur but also achieves sustained macrophage-targeted drug release, with the release rate increasing with H_2_O_2_ concentration. In vivo experiments demonstrated that the HASF@Cur nanomicelle group showed a reduction in aortic plaque area (38.0 ± 4.9%,) compared to the free Cur group (22.1 ± 4.5%,), indicating superior targeting specificity and therapeutic effectiveness of this drug delivery system for AS treatment [15]. The inorganic NDDS Cur-MnO_2_/HA, which encapsulates the drug Cur, is composed of HA, MnCl_2_, and NaOH. The results of in vitro experiments show that the mesoporous structure and abundant metal coordination sites in MnO_2_ contribute to a high drug loading capacity of 54%, while the surface-modified HA specifically binds to the CD44 receptor expressed by peri-macrophages in AS lesions to achieve precise targeting (Figure 1A). In an ApoE^−/−^ mouse model, this NDDS extended the circulating half-life of Cur by 6-fold and increased drug accumulation at the AS lesion site by 3.5-fold, ultimately reducing plaque area and lipid levels [16]. The above nano-delivery systems have improved the utilization of Cur in different ways through their unique design and mechanism of action, which enhance the performance of Cur and overcome the problems in the traditional application of Cur, which is conducive to the research and development of AS therapy.

#### 2.1.2. Quercetin (QT)

QT is a flavonoid compound commonly found in the daily diet and in some Chinese herbal medicines, including vegetables, fruits, and teas, with antioxidant, anti-inflammatory, antiproliferative, anticancer, antidiabetic, and antiviral properties [18,19]. However, the abundance of phenolic hydroxyl groups brings certain challenges, rendering QT susceptible to degradation, limiting its stability, hampering its bioavailability due to high polarity, and thus restricting its application in disease prevention [20]. Macrophages play a central role in the pathologic process of AS, and their functional abnormalities are present throughout the development of the disease. In the treatment of AS with QT, macrophages are not only key cells regulated to suppress disease progression but also serve as carriers for the targeted delivery of drugs to lesion sites. QT itself can suppress the progression of AS by regulating MST1-mediated autophagy in oxidized low-density lipoprotein (ox-LDL)-induced RAW264.7 macrophage foam cells [21]. In the biomimetic NDDS MP-QT-NP, QT-loaded liposomes are attached to the macrophage surface via β-cyclodextrin (β-CD)-mediated host–guest interaction, enabling macrophages to act as targeted drug carriers for precise delivery to AS plaques. In vivo experiments indicate that the accumulation of MP-QT-NP in the aortas of ApoE^−/−^ mice was 3.9 times higher than that of free QT-NP (*p* ≤ 0.05). Moreover, due to the camouflage strategy of MP-NP conjugation, the overall blood circulation half-life of MP-Cy5-NP was extended to 13.1 h, compared to 3.86 h for free Cy5-NP and 3.92 h for MP + Cy5-NP. This delivery system enhances the water solubility of QT, promotes cholesterol dissolution via β-CD, exerts local anti-inflammatory effects through QT, and synergistically activates the LXR and NRF2 pathways [22].

#### 2.1.3. Baicalin (BC)

BC is one of the active ingredients in the skullcap, with a variety of pharmacological effects, such as blood pressure reduction, sedation, liver protection, gallbladder protection, anti-bacteria, and anti-inflammation [23]. In the prevention and treatment of AS, BC can mitigate AS by reducing inflammation-induced damage, regulating lipid metabolism, promoting cell survival, and modulating immune responses [24]. Due to its low bioavailability, emerging novel baicalin preparations including nano/micro-scale baicalin delivery systems show better absorption and higher bioavailability in preclinical studies, and show promise for future clinical applications [25]. For instance, dual-targeted pH-responsive NDDS BC@CS/cRGD NPs convert natural drug BC into efficient targeted formulations. This is achieved by combining BC with chondroitin sulfate (CS) via an amidation reaction, followed by the modification of the targeting peptide cRGD to target the integrin α_v_β_3_ receptor overexpressed on the surface of endothelial cells and macrophages. The acidic environment of the AS plaque microenvironment causes the amid bond to break at low pH, enabling controlled release of BC. In vivo experiments indicate that the fluorescence accumulation intensity of Cy5-labeled BC@CS/cRGD NPs in aortic plaques of AS model mice is 1.19-fold (*p* < 0.05) and 1.24-fold (*p* < 0.05) higher than that of the non-targeting group, respectively. The dual-targeting strategy effectively targets both endothelial cells and macrophages, disrupting the vicious cycle of inflammation-induced endothelial injury. pH-triggered release enhances the local drug concentration in the plaque, promoting anti-inflammatory, endothelial repair, lipid-lowering, and multifunctional synergistic effects [26].

#### 2.1.4. Resveratrol (RSV)

RSV is a natural phenolic compound found in a variety of foods such as grapes, peanuts, blueberries, red wine, etc., which possesses a variety of biological activities such as antioxidative, anti-inflammatory, immunomodulatory, blood-pressure-lowering, and lipid-lowering activities, and can be used in the treatment of diseases such as CVD and obesity [27]. RSV is also a cardioprotective phytoalexin, which can hinder oxidative damage by affecting the superoxide and hydroxyl radical anions, preventing ROS formation and lipid peroxidation [28]. Furthermore, RSV counteracts inflammation in human M1 and M2 macrophages upon challenge with 7-Oxo-Cholesterol, indicating potential therapeutic implications in AS [29]. In addition, spontaneous encapsulation of macrophage membranes, modification with the peptide CLIKKPF and loading with the hydrophobic compound RSV allow the construction of bionic nanoparticles MM@NPs (Figure 1B), whose surface macrophage membrane coating could help the nanoparticles evade immune clearance. Integrin α4β1 retained on the surface of macrophage membranes can interact specifically with activated endothelial cells highly expressing VCAM-1 to promote targeted delivery to inflammatory lesions. In vitro results showed that MM@NPs were taken up by activated endothelial cells, reduced macrophage ROS levels, and inhibited ox-LDL-induced foam cell formation. MM@NPs integrated multiple functions to prolong circulation time and selectively target plaques to increase the local drug concentration, thereby enhancing its therapeutic effect [17].

### 2.2. Terpenes and Their Derivatives

#### 2.2.1. Celastrol (Cel)

Cel is an active pentacyclic triterpenoid extracted from *Tripterygium wilfordii* and has anti-inflammatory and anti-tumor properties and inhibits platelet function and venous/arterial thrombosis [30]. In terms of metabolic regulation, it can ameliorate lipid metabolism disorders by modulating lipid profiles and related metabolic processes and has a strong lipid-modulating ability [31]. However, the safety issue of Cel is a major obstacle to its clinical translation. For in vivo animal systems, the LC_50_ (50% lethal concentration) values of Cel against 48 h post fertilization zebrafish embryos and adult rats were 0.9 μM and 12.5 μg/g/d [32]. For this purpose, researchers developed Cel-loaded poly (ethylene glycol)-b-poly (propylene sulfide) (PEG-b-PPS) micelles. The NDDS encapsulates Cel through its hydrophobic core PPS, while taking advantage of the targeted release achieved by the oxidation-sensitive property of PPS. This strategy enabled a 10,000-fold increase in Cel solubility in 1xPBS in the nanomicelles and also addressed toxicity issues by reducing the effective dose. In terms of therapeutic efficacy, in vivo studies show that the group treated with NDDS showed better efficacy than the free drug, with a significant reduction in plaque area compared to the blank-micelle group, whereas the free Cel did not show this effect [33].

#### 2.2.2. Paclitaxel (PTX)

PTX is a tricyclic diterpenoid compound naturally produced in the bark and needles of *Taxus brevifolia* [34]. It is used as an important tumor therapeutic agent, and its cytotoxicity can hyperstabilize microtubules against depolymerization and, consequently, arrest cell division [35]. Studies have shown that the toxicity of PTX is significantly reduced when it is combined with cholesterol-core nanoparticles (LDE) [36]. Therefore, combining LDE with PTX may provide a new, safer and more efficient drug delivery strategy for the treatment of AS. For example, lipid-based NDDS encapsulating the drug PTX showed efficacy. In vivo experiments showed that LDE-PTX treatment resulted in a reduction in wall area by 14% and stenosis by 22%. The body weight remained unchanged in all groups, which indicates that there was no significant toxicity in the LDE-PTX group [37]. Given the potential already demonstrated by the combination of PTX with LDE particles in reducing toxicity and improving therapeutic efficacy, it is expected that in the future, its unique uptake mechanism will be explored in depth, and the combination will be further optimized to broaden the scope of its application in the treatment of tumors and diseases such as AS.

#### 2.2.3. 1,8-Cineol (CIN)

CIN is a natural organic monoterpene compound mainly found in eucalyptus, rosemary, tea tree, bay leaf and other aromatic plants, which has shown potential applications in medicine-related fields due to its anti-inflammatory, antibacterial and antioxidant effects [38,39]. Currently, CVD treatment mainly relies on long-term drug interventions; however, the difficulty in achieving precise and selective targeting of active ingredients often leads to toxic reactions or other complications, highlighting advantages of enhanced targeting by nanodelivery technologies. For example, in the oral nanoemulsion CIN@DEX_5k_-BSA/PTM/VB_12_, which is loaded with CIN, Dextran (DEX) targets macrophages in AS plaques, and Vitamin B12 (VB_12_) promotes intestinal absorption. Bovine serum albumin (BSA) anchors the oil phase through the hydrophobic region and exposes the hydrophilic region to the aqueous phase to form a stable oil-water interface. The overall improved stability of CIN in vitro and in vivo prolonged the retention time in the gastrointestinal tract, while enhancing the permeability of the mucus layer and intestinal epithelial cells, which resulted in improved oral bioavailability and plaque accumulation of CIN. In vivo studies showed that free CIN resulted in only a slight reduction in the stained area of the aortic root, whereas the use of CIN delivered in the form of nanoemulsions enhanced its therapeutic efficacy in AS [40]. In addition, researchers have developed MM-CIN-BDS, a biomimetic NDDS for CIN with MM and diethylaminoethyl-dextran (DEAE) modification targeting endothelial cells at sites of inflammation via monocyte (MM) membranes. MM binds to venous endothelial cells via VLA-4/VCAM-1 interaction and migrates to the vascular media via CD31/PECAM-1 interaction, which can be effective in targeting AS lesions. In vivo experiments indicate that MM-CIN-BDS treatment reduced the aortic lesion area in mice with a dynamic AS model, and its efficacy was close to that of statins [41].

#### 2.2.4. Tanshinone IIA (TanIIA)

TanIIA is a compound extracted from the traditional herb *S. miltiorrhiza*, which has antioxidant and anti-inflammatory properties [42,43]. TanIIA protects vascular smooth muscle cells, improves endothelial cell dysfunction and inhibits macrophage inflammatory response and foam cell formation in AS plaques, etc. [44]. TanIIA is hydrophobic but lipophilic when used alone, meaning it has disadvantages in utilization, while combining it with lipids can effectively improve its solubility and stability, as demonstrated by the lipid-based NDDS loaded with TanIIA, pHDL-TanIIA, in which the hydrophobic component of TanIIA was embedded with peptide-based high-density lipoprotein (pHDL) using microfluidic technology to obtain good water solubility characteristics. In vivo studies show that pHDL-TanIIA achieved a 36.86% reduction in plaque area, outperforming both pHDL alone (28.5% reduction) and free TanIIA (21.96% reduction) [45]. This lipid-based drug delivery strategy not only overcomes the challenges of poor solubility and instability associated with hydrophobic compounds but also provides a promising solution to improve the clinical applicability of hydrophobic compounds.

### 2.3. Alkaloid

#### 2.3.1. Colchicine (COL)

COL is an active ingredient isolated from the plant *Colchicum autumnale* with anti-inflammatory effects and potential therapeutic benefits for various diseases [46]. It modulates leukocyte trafficking in AS and reduces vascular inflammation [47]. However, excessive does of COL may cause severe systemic toxicity including gastrointestinal disorders and multi-organ failure. To overcome this limitation, researchers developed a polymer-based NDDS, VHPK-PLGA@COL, which encapsulates COL to optimize dosing while minimizing adverse effects. The system features a VHPK peptide targeting VCAM-1 on endothelial cells, poly (lactic-co-glycolic acid) (PLGA) extending half-life, and poly (ethylene glycol) (PEG) avoiding reticuloendothelial clearance while prolonging circulation. The combined effects of sustained release and targeted delivery reduce nonspecific uptake, leading to lower cytotoxicity compared to free COL. In vivo studies demonstrated that while both free COL and VHPK-PLGA@COL exhibit anti-AS effects, the VHPK-PLGA@COL group showed superior outcomes, including a minimal plaque burden, the most reduced lipid deposition and the least macrophage infiltration when compared to both COL-treated and untreated groups [48].

#### 2.3.2. Berberine (BBR)

BBR, a botanical alkaloid present in the roots and bark of various plants, serves as the primary active component of *Rhizoma coptidis*—a traditional Chinese herb extensively employed in treating diabetes and infectious diseases [49]. Research demonstrates that chronic BBR administration in ApoE^−/−^ mice not only attenuates arterial lesions but also suppresses aortic oxidative stress and adhesion molecule expression, while upregulating UCP2 levels [50]. However, its clinical application is limited by extremely low oral bioavailability (<1%). To overcome this, researchers developed a polymer-based NDDS termed BBR NPs@Man/M2, where (1) PLGA nanoparticles enable BBR sustained release; (2) mannose modification targets macrophage surface mannose receptors for inflammatory site accumulation; and (3) M2 macrophage membrane coating confers both immune evasion and inflammatory targeting capabilities. In vitro studies showed that the M2 membrane functionalization facilitated effective macrophage targeting in vitro, inducing phenotypic transition from pro-inflammatory to anti-inflammatory states. This polarization not only alleviated endothelial inflammation but also enhanced repair mechanisms in damaged endothelial cells [51].

### 2.4. Miscellaneous

#### 2.4.1. Geniposide (GP) and Emodin (EM)

GP, an iridoid glycoside derived from *Gardenia jasminoides* J., exhibits multiple pharmacological properties including antidiabetic, hepatoprotective, anti-inflammatory, analgesic, and cardioprotective effects [52]. Gardenia glycosides can inhibit the progression of AS by regulating macrophage activity. The mechanisms involve enhancing macrophage autophagy to reduce plaque formation. This is achieved by down-regulating TREM2. Additionally, gardenia glycosides can ameliorate AS by affecting macrophage polarization, which is achieved through regulating perivascular adipocyte-derived CXCL14 [53,54].

EM is a naturally occurring anthraquinone derivative found in various TCM plants, exhibiting diverse pharmacological activities including anticancer, anti-inflammatory, antioxidant, antibacterial, antiviral, antidiabetic, and immunosuppressive effects [55,56]. In the context of AS, EM has been shown to suppress NLRP3/GSDMD-mediated inflammation through inhibition of the TLR4/MyD88/NF-κB signaling pathway, thereby attenuating AS plaque progression [57]. Furthermore, studies demonstrate that emodin-loaded nanocapsules M-CS-E-LNC can inhibit acute pancreatitis by regulating lipid metabolism reprogramming during macrophage polarization [58].

**Figure 2:**
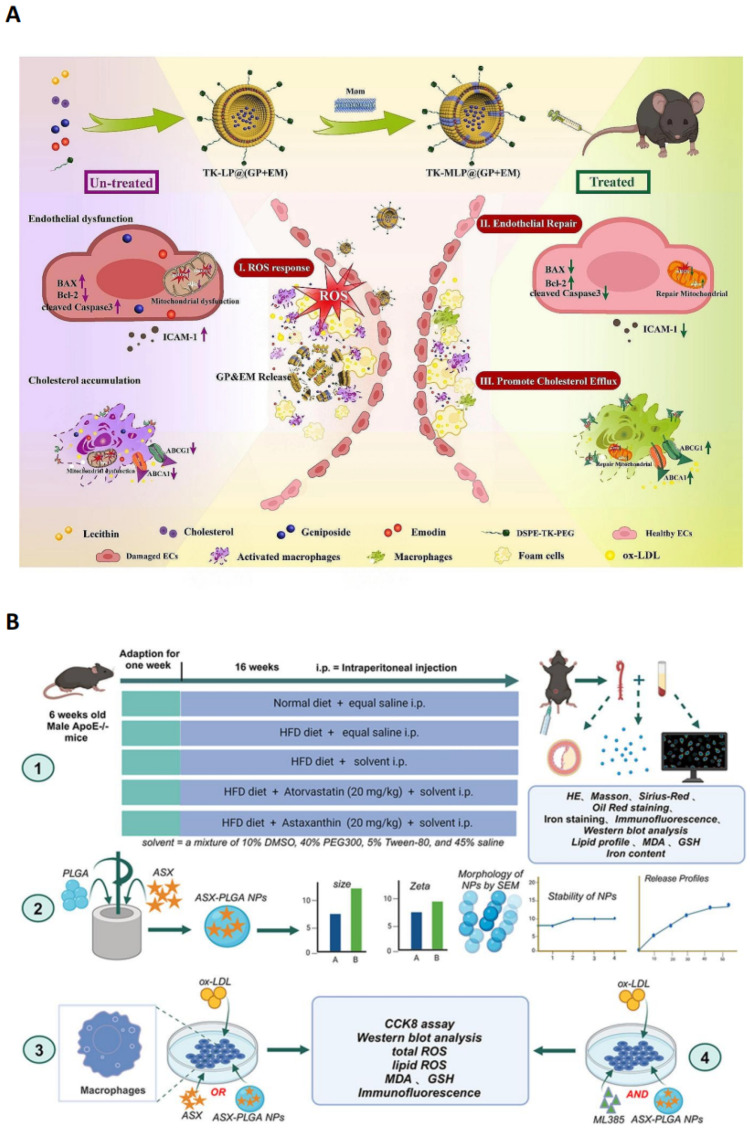
(**A**) Illustration of the preparation of TK-MLP@ (GP  +  EM) NPs and the strategy for atherosclerosis treatment. Reproduced with permission [59]. Copyright 2024, *Journal of Nanobiotechnology*. (**B**) Schematic diagram depicting how astaxanthin-loaded polylactic acid–glycolic acid nanoparticles alleviate AS. Reproduced with permission [60]. Copyright 2025, the authors. Published by Elsevier Inc.

To fully exert the synergistic effects and enhance bioavailability of GP and emodin EM, researchers developed a macrophage membrane (Møm) hybrid NDDS. The system utilizes macrophage membrane (Møm) and TK to encapsulate GP and EM to form nanoparticles TK-MLP@ (GP + EM) NPs (Figure 2A), which achieves synergistic delivery with dual therapeutic mechanisms. GP is mainly responsible for repairing the damaged endothelial barrier and blocking the “entrance” of lipid infiltration, while EM promotes cholesterol efflux from the macrophage to unclog the “exit” of lipid clearance. In vivo studies show that this biomimetic NDDS has the significant advantage that Møm membrane camouflage enables a 1.91-fold increase in nanoparticle enrichment at the plaque site and long circulation, while the ROS-responsive thioketal (TK) system ensures the precise release of the drug at the lesion site. The inhibitory effect of TK-MLP@ (GP + EM) NPs on aortic plaque formation is stronger than that of the free drug combination, and the combinatorial effect is superior to single-drug treatment [59].

#### 2.4.2. Artemisinin (ART) and Proanthocyanidins (Pc)

ART, a sesquiterpene lactone derived from the traditional Chinese herb *Artemisia annua* L., can be used in the treatment of cancer, febrile diseases, diabetes mellitus, and so on [61]. It was discovered by Professor Youyou Tu, who was awarded the Nobel Prize in Physiology or Medicine in 2015 for this discovery [62]. However, it is poorly soluble in water and oil [63]. ART attenuates the development of AS lesions by the regulation of vascular smooth muscle cell phenotype switching [64]. It also alleviates atherosclerotic lesion by reducing macrophage inflammation via regulation of AMPK/NF-κB/NLRP3 inflammasomes pathway [65].

Pc is a polyphenol compound that is widely distributed in the bark, fruit core, skin, or seeds of various plants and possesses functions such as antioxidation, cardioprotection, immunomodulation, lipid-lowering and antidiabetic effects [66,67]. Pc-rich fractions obtained from the bark of *Croton celtidifolius* Baill prevent isolated LDL oxidation, decrease oxidative stress in endothelial cells and improve endothelial function in mice with cardioprotective effects [68].

Both ART and Pc demonstrate antioxidant properties with promising therapeutic potential for AS. Researchers have combined ART and Pc to construct the biomimetic HybridNDDS HA-M@PB@ (PC + ART) coated with macrophage–erythrocyte hybrid membranes and modified with HA. The membrane coating retains the inflammatory chemotactic property of macrophage membrane and the long-circulating property of the erythrocyte membrane. Moreover, HA modification targets the activated macrophages within the plaque via the CD44 receptor. The improvement of the biocompatibility and immune evasion ability of this NDDS reduces the amount of drug clearance during the circulation process. In vitro results show that the prepared nanocomplex has a significant scavenging effect on ROS and nitric oxide (NO). Secondly, it can inhibit the NF-κB/NLRP3 pathway, leading to the inhibition of lipid influx. In this way, it can achieve the controlled release of drugs in inflammatory macrophages and alleviate AS lesions [69].

#### 2.4.3. Vitamin C (VC)

VC also known as L-ascorbic acid is a nutrient naturally occurring in many fruits and vegetables that has potent antioxidant activity but cannot be synthesized by the body itself [70,71]. Studies have shown that VC deficiency can exacerbate coronary artery dysfunction in AS fed a high-fat diet [72]. However, natural VC has problems such as limited ROS elimination capacity and rapid metabolism; for this reason, natural antioxidant VC was loaded into the natural antioxidant lipoic acid (LA)-constructed cross-linked vesicles to develop nanomedicine VC@cLAVs. Due to the tight cross-linked disulfide core and negative carboxylic-acid-enriched surface, the VC@cLAVs effectively avoided blood blood dilution and serum protein adhesion, greatly increasing the blood half-life of natural antioxidants. This NDDS formed LA/DHLA and VC/DHA redox cycles after entering cells, regenerating each other and continuously scavenging ROS, amplifying the antioxidant capacity. In vitro studies demonstrated that the half-life of VC@cLAVs was 7.3-fold and 10.2-fold higher than that of free VC and LA, respectively, and the intracellular ROS elimination rate was 83%, which was 6.7-fold and 4.2-fold higher than that of VC and LA, respectively, and effectively overcame the rapid metabolism of VC and LA. Moreover, in vivo results demonstrated that VC@cLAVs reduced the plaque area of ApoE^−/−^ from 52% to 13%, and the therapeutic effect was much higher than that of free VC and LA [73]. Extracellular vesicles , which are phospholipid bilayer membrane structures secreted by cells, serve as an important basis for discovering potential diagnostic biomarkers and therapeutic targets for AS [74]. Their potential application in the field of NNDS in the future constitutes a hypothesis worth exploring.

#### 2.4.4. Astaxanthin (ASX)

Astaxanthin, a lipid-soluble carotenoid produced by various microorganisms and marine animals, possesses antioxidant, anti-inflammatory and anti-apoptotic properties, enabling it to prevent and treat various diseases [75]. With its potent antioxidant properties (reducing oxidative stress and enhancing the bioavailability of NO) and anti-inflammatory characteristics (inhibiting the NF-κB/MAPK pathway and promoting the reverse cholesterol transport), astaxanthin is expected to become an effective pharmaceutical drug for the prevention and treatment of cardiovascular diseases [76]. However, ASX, as a highly fat-soluble carotenoid, exhibits poor oral bioavailability; for this reason, researchers developed polymer-based NDDS-ASX-PLGA NPs (Figure 2B). PLGA endows ASX with better solubility and stability. Studies have shown that ASX inhibits AS by inhibiting macrophage iron death, regulating oxidative stress and activating the NRF2 pathway. In addition, the therapeutic effect of ASX against AS showed high similarity to that of statins and was superior to that of free ASX monotherapy [60].

In summary, in vitro and in vivo experiments have confirmed that BNP-based NDDSs effectively inhibit inflammatory responses, lipid deposition, and plaque formation during atherosclerosis by optimizing drug targeting, stability, and bioavailability (Table 1), providing innovative strategies with both safety and efficiency for clinical AS treatment.

Notably, the advancements in these NDDS exemplify the growing potential of nano-delivery technologies in disease therapy. In recent years, rational design of nanoparticle composition, structure, and functionality has enabled efficient synergies among various therapeutic modalities. Below is a summary of the essential characteristics and delivery strategies of the NDDS discussed in this paper (Table 2 and Table 3), aiming to provide insights for the future development of nanomedicines.

## 3. Natural Compounds with Potential for Treating AS

BNPs have demonstrated promising potential in the prevention and treatment of cardiovascular diseases. Their antioxidant, anti-inflammatory and lipid-regulating activities prove useful in drug discovery [81,82]. The deployment of nano-delivery systems to encapsulate BNPs constitutes a feasible strategy for enhancing their bioavailability and therapeutic efficacy. This synergistic integration is increasingly recognized as a valuable paradigm in cardiovascular therapy [83]. We selected some natural products that exert beneficial effects against atherosclerosis (AS), aiming to provide a reference framework for the rational design of related therapeutic agents.

### 3.1. Antioxidant and Anti-Inflammatory Compounds

Inflammation is a core driver of AS, involving dyslipidemia and dynamic interactions between immune and vascular cells [84,85]. Oxidative stress promotes the adhesion and infiltration of monocytes into the vascular intima by generating oxidized ox-LDL and activating inflammatory signaling pathways such as NF-κB, thereby creating a chronic inflammatory environment [86]. AS inflammation is regulated by multiple signaling pathways. Pro-inflammatory pathways like NF-κB, which activates the NLRP3 inflammasome by the TLR-MyD88 signaling in response to damage signals, form an NF-κB-NLRP3 cascade activation network [87,88]. The NLRP3 inflammasome in AS recognizes cholesterol crystals to activate caspase-1, which cleaves pro-IL-1β and pro-IL-18 into their active forms, exacerbating the inflammatory response [89]. STAT3 plays a key role in cell growth and apoptosis, regulating the expression of cytokines, chemokines, growth factors, and collaborating with NF-κB to form and maintain the inflammatory microenvironment [90]. Anti-inflammatory pathways such as AMPK can reverse and regulate macrophage function to form an anti-inflammatory phenotype and participate in anti-AS processes through endothelial protective effects, with induction of SIRT1 activation being an important mechanism [91,92,93]. Modulating these pathways has become a potential therapeutic strategy and various natural compounds have shown regulatory effects on the above inflammatory signaling pathways (Table 4).

#### 3.1.1. Salidroside (SAL)

SAL has been identified as one of the most potent compounds isolated from various *Rhodiola* plants, with most of the global species distributed in China [110]. Recent studies show that SAL exerts anti-inflammatory and antioxidant effects through multiple mechanisms, reducing pro-inflammatory cytokine expression while increasing anti-inflammatory cytokine expression. SAL inhibits the NF-κB signaling pathway and has been shown to protect endothelial cells by activating AMPK and suppressing the NF-κB p65/NACHT, LRR, and NLRP3 signaling pathway [94]. Additionally, SAL activates the AMPK/sirtuin-1 (SIRT1) pathway, reducing MDA levels and increasing SOD levels in human umbilical vein endothelial cells (HUVECs) in vitro [111]. In ApoE^−/−^ mouse models, SAL inhibits caspase-1 activation and IL-1β release at a dose of 50 mg/kg, thereby reducing NLRP3-related pyroptosis [95]. SAL exhibits a wide range of biological activities, including in the cardiovascular and nervous systems, but its targeting is insufficient [112]. Its anti-inflammatory effects on multiple systems may lead to immune imbalance and potential risks. SAL’s bioavailability varies significantly with different administration methods. For instance, in rat models administered 12 mg/kg orally, the bioavailability of SAL was 32.1%, while in rats given 25 mg/kg orally or 5 mg/kg intravenously, the bioavailability reached 98% [113,114]. We believe that the use of NDDS to administer this drug can enhance its bioavailability, thereby enabling its anti-inflammatory and antioxidant effects to be targeted specifically at arterial plaque regions.

#### 3.1.2. Luteolin (LUT)

LUT, a polyphenolic compound found in numerous natural plants, possesses a wide array of pharmacological activities [115,116]. It demonstrates anti-inflammatory, antioxidant, anti-apoptotic, and vascular-protective effects, showing potential in AS treatment. Studies have indicated that LUT can inhibit STAT3 activation, reducing the expression of inflammatory factors in AS mice, such as ICAM-1, VCAM-1, IL-6, and TNF-α, and curbing AS progression. In LUT-treated groups, the plaque area in ApoE^−/−^ mice was reduced by approximately 50% [97]. Other research has shown that LUT can modulate the SIRT1/CXCR4 signaling pathway, dose-dependently increasing SOD activity and decreasing MDA activity in SD rats. It also downregulates the expression of caspase-3 and caspase-9 while upregulating Bcl-2 expression, thereby inhibiting oxidative stress and enhancing autophagy to alleviate vascular calcification [96]. However, studies have shown that LUT primarily exists in the form of glucuronides and sulfates within the body, which may limit its bioactivity. The use of NDDSto administer this drug may increase its plasma concentration and improve bioavailability [98].

### 3.2. Compounds That Regulate Cell Transformation and Proliferation

AS lesions can damage vascular endothelial cells, and such endothelial dysfunction is closely related to the formation and progression of vulnerable plaques [117]. Endothelial-to-Mesenchymal Transition (EndMT) is a significant manifestation of endothelial dysfunction, promoting vulnerable plaque formation [118]. During AS progression, vascular smooth muscle cells (VSMCs) undergo migration and proliferation. Such migration is associated not only with overall plaque development but also with the life cycle and specific phenotypes of VSMCs [119]. Recent perspectives suggest that VSMCs exhibit diverse origins and phenotype-related functions. Their proliferation may play a reparative and plaque-stabilizing role throughout the disease process, while VSMC apoptosis and senescence can disrupt the plaque fibrous cap, increasing plaque instability [84]. MicroRNAs (miRNAs) contribute to regulating the inflammatory state of vascular endothelial cells, as well as the proliferation, senescence, and phenotypic transition of smooth muscle cells. Due to these multifaceted roles, miRNAs present themselves as highly promising candidates for innovative therapeutic targeting strategies [120].

#### 3.2.1. Icariin (ICA)

ICA, a bioactive component of the traditional Chinese herb Epimedium, has recently been reported to exert anti-AS effects by regulating EndMT, VSMC migration, and ferroptosis. ICA primarily modulates miRNAs to influence EndMT and VSMC migration and proliferation. Studies have shown that ICA regulates the H19/miR-148b-3p/ELF5 axis to inhibit EndMT. Experimental data indicated that ICA treatment upregulated H19 expression, suppressed miR-148b-3p expression, and simultaneously increased ELF5 expression at both mRNA and protein levels, effectively blocking the ox-LDL-induced EndMT process [100]. In terms of VSMC migration, ICA downregulates miR-205-5p expression, activates the ERBB4/AKT signaling pathway, and inhibits ox-LDL-induced proliferation and migration [99]. A team combined ICA with methoxypolyethylene glycol (mPEG) to prepare nanoparticles to improve myocardial ischemia. This formulation effectively addressed the poor water solubility of ICA, thereby enhancing its bioavailability [121].

#### 3.2.2. Evodiamine (EVO)

EVO is a quinolone alkaloid derived from the fruit of Evodia rutaecarpa. This herb exhibits a broad spectrum of pharmacological activities and holds applications for numerous chronic conditions [122]. The TCM formula Wu-Zhu-Yu Decoction (WZYD), which features Evodia rutaecarpa as a core herb, exhibits anti-AS properties. It reduces plaque area in HFD-induced ApoE^−/−^ mice. In high-dose groups, the plaque area was reduced by approximately 84.2% compared to the model group, while also inhibiting VSMC proliferation and migration [123]. Multiple studies have indicated that EVO regulates VSMC proliferation and migration through mechanisms such as inhibiting the PI3K/Akt signaling pathway and activating PPARγ, showing potential in AS treatment [101,102]. Additionally, ABCA1 is a direct binding target of EVO, and EVO regulates cholesterol metabolism by modulating ABCA1 expression [124]. However, Evodia rutaecarpa exhibits hepatotoxicity, with studies showing that EVO can dose-dependently reduce cell activity [103]. A team developed a novel water-in-oil nanoemulsion containing evodiamine-phospholipid nanocomplex (NEEPN), which enhanced the oral bioavailability of EVO, reaching a relative bioavailability of 630.35%. This formulation also reduced the liver first-pass effect, prolonged drug action time, and thus helped to mitigate potential toxicity [125]. Consequently, we posit that employing a nano-delivery system as a carrier for EVO (everolimus) holds the potential to amplify its therapeutic efficacy while simultaneously mitigating toxicity, thereby paving the way for safer and more clinically effective applications.

### 3.3. Compounds That Regulate Lipid Metabolism

The pathological progression of AS is closely linked to lipid metabolism disorders. The oxidation and subsequent deposition of LDL within the vascular intima represent a primary inciting event. It recruits monocytes to infiltrate the intima, where they differentiate into macrophages. These macrophages take up large amounts of ox-LDL through the lectin-like oxidized low-density lipoprotein receptor-1 (LOX-1), eventually forming foam cells, which constitute the core components of AS plaques [126]. Lipid metabolism disorders not only drive the expansion of the lipid core within plaques but also accelerate lesion progression by activating inflammatory responses [85]. An increase in ATP-binding cassette transporter A1 (ABCA1) expression can regulate membrane protein distribution, promote cholesterol efflux from macrophages, and enhance reverse cholesterol transport (RCT), thereby exerting anti-AS effects [127]. In recent years, natural compounds targeting lipid metabolism have become a research hotspot. However, their clinical application is limited by issues such as low bioavailability and poor targeting.

#### 3.3.1. Leoligin (LEO)

LEO, a kind of lignan in Edelweiss, which can promote cholesterol reverse transport to regulate lipid metabolism. Studies have confirmed that LEO activates CETP in rabbits and ApoE^−/−^ mouse models to facilitate cholesterol transport. Research has also shown that LEO upregulates the protein levels of ABCA1 and ABCG1, promoting cholesterol efflux from THP-1 macrophages and enhancing cholesterol outflow mediated by human plasma, indicating potential for preventing or treating AS-related diseases [104,105]. However, LEO has been reported to inhibit the proliferation of endothelial cells, which may affect vascular endothelial repair and regeneration, suggesting potential toxicity. This indicates that LEO could be delivered via a nanodelivery system to achieve detoxification and enhanced efficacy [106].

#### 3.3.2. Oridonin (ORI)

ORI is a diterpenoid compound from the traditional Chinese herb Rabdosia rubescens, which has demonstrated potential in treating cardiovascular diseases. Its anti-atherosclerotic (anti-AS) effects mainly involve lipid metabolism regulation and inflammation inhibition [128]. Its mechanisms may involve modulating the FABP4/PPARγ signaling pathway and downregulating CD36 expression. Animal experiments have shown that ORI increases ABCA1 expression and inhibits lipid accumulation within cells in ApoE^−/−^ mice models [107,108]. However, the clinical application of ORI is limited by its pharmacokinetic properties. Studies have indicated that ORI has poor bioavailability. When administered orally to rats at doses of 20, 40, and 80 mg/kg, the absolute bioavailability of ORI was 4.32%, 4.58%, and 10.80%, respectively [109]. A team has constructed RGD-modified polylactic acid nanoparticles, which effectively enhance the bioavailability of ORI and strengthen its targeting of tumor tissues [129]. Based on existing evidence, developing an actively targeted nano delivery system may be an effective strategy to overcome the bioavailability limitations of ORI and achieve precise treatment of arterial plaques.

## 4. Challenges

While the integration of BNPs with NDDS has shown promising results in preclinical studies for AS treatment, several critical challenges remain that hinder their clinical translation. These challenges warrant in-depth discussion to guide future research directions.

### 4.1. Nanoparticle Toxicity Concerns

Notably, some BNPs inherently possess toxicity (e.g., celastrol’s lethal effects in zebrafish embryos [32] and evodiamine’s hepatotoxicity [103]), and while NDDS can reduce effective doses, residual toxicity risks persist. For example, the PLGA-based delivery system for colchicine (VHPK-PLGA@COL) mitigates systemic toxicity but requires long-term monitoring of polymer degradation byproducts in humans [48].

### 4.2. Regulatory Hurdles

The regulatory approval of nanoformulations is significantly more complex than conventional drugs, primarily due to the unique physicochemical properties of NPs (e.g., size, surface charge, and aggregation behavior) that affect their biodistribution and safety [10]. Regulatory agencies such as the FDA and EMA require extensive characterization of NP stability, batch-to-batch consistency, and long-term toxicology data—standards that are often challenging to meet for novel NDDS.

### 4.3. Stability Limitations

Both BNPs and their nanoformulations face stability challenges. Many BNPs (e.g., curcumin, resveratrol) are prone to oxidation or hydrolysis under light, heat, or acidic conditions [14,27]. While NDDS like Cur-MnO_2_/HA NPs improve stability via encapsulation [16], long-term storage remains problematic: lipid-based carriers (e.g., liposomes) often suffer from phase separation, and polymeric micelles may aggregate in aqueous solutions over time [45].

In vivo stability is equally critical. NPs can interact with serum proteins (opsonization) or be rapidly cleared by the reticuloendothelial system (RES), reducing circulation time. For example, unmodified quercetin NPs are cleared within 4 h in mice, whereas macrophage membrane-camouflaged MP-QT-NP extends circulation to 13.1 h—but such camouflage strategies add complexity to formulation design [22].

### 4.4. Preclinical-to-Clinical Gaps

Despite promising preclinical results, translating NDDS to human trials remains challenging. Animal models (e.g., ApoE^−^/^−^ mice) recapitulate key AS features but fail to mimic human disease complexity, including aging, comorbidities (e.g., diabetes), and genetic diversity [84]. Dose extrapolation is another hurdle: effective doses in mice (e.g., 50 mg/kg for salidroside [95]) may not scale linearly to humans, risking underdosing or toxicity.

Moreover, preclinical studies often use healthy animals or idealized disease models, whereas clinical AS patients exhibit heterogeneous plaque phenotypes (e.g., stable vs. vulnerable plaques). This discrepancy may explain why some nanoformulations (e.g., LDE-PTX NPs [37]) show dramatic plaque reduction in mice but require re-evaluation in patient subgroups.

## 5. Conclusions and Future Perspectives

Beyond summarizing existing evidence, our work highlights a notable pattern: the majority of BNPs with well-characterized pharmacological mechanisms discussed in this review originate from TCM. The dominance of TCM-derived examples reflects their unique historical and scientific value. TCM’s 2000+ year empirical legacy provides a robust foundation for characterizing its herbs that contain diverse bioactive compounds such as ginsenosides and tanshinones, with well-documented mechanisms including anti-inflammatory and cardioprotective effects. Additionally, their structural diversity and strong translational momentum, including increasing clinical trials and regulatory approvals, have driven intensive research, naturally leading to their prominent role in the literature. This enables efficient synergy of multiple therapeutic approaches, fully demonstrating the potential of nanodelivery technology in AS treatment. To advance clinical translation, future research can focus on scalable manufacturing, rigorous clinical validation, advanced bionic nanocarriers and personalized nanomedicine. In summary, while challenges in toxicity, regulation and clinical translation persist, the synergy between BNPs and NDDS holds immense potential to revolutionize AS therapy, offering safer, more effective, and personalized treatments for cardiovascular disease.

## Figures and Tables

**Table 1:** In vivo and in vitro experiments of BNPs with NDDS for AS treatment.

Natural Drug Monomer	Drug Molecular Formula	Structure Diagram	NDDS	Animal Model	Target	Drug Release	Signaling Pathway	Role of NDDS in Drug Efficacy	Experimental Outcome	References
Cur	C_21_H_20_O_6_	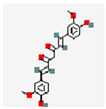	HASF@Cur	Male rat	Macrophage CD44 receptor	ROS-responsive controlled release	NF-κB	Localize Cur release; enhance Cur bioavailability	Reduced AS plaque area	[15]
			Cur-MnO_2_/HA	ApoE^−/−^ mice	M1 macrophages	pH and GSH concentration-controlled release	HIF-1α	Extend Cur circulation half-life by 6-fold; enhance bioavailability	Alleviate oxidative stress Suppress inflammation	[16]
BC	C_21_H_18_O_11_	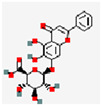	BC@CS/cRGD NPs	ApoE^−/−^ mice	Macrophages	Rapid release in acidic conditions	TLRs/NF-κB p65	Rapid drug release and enhance drug accumulation	Protects endothelial cells	[26]
RSV	C_14_H_12_O_3_	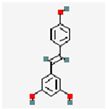	MM@NPs	ApoE^−/−^ mice	Activated endothelial cells; inflammatory macrophages	pH-responsive controlled release		Efficient RSV encapsulation; targeted delivery; mitigates toxic side effects	Antioxidant, anti-inflammatory and formation-inhibiting effects	[17]
Cel	C_29_H_38_O_4_	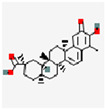	Cel-loaded PEG-b-PP micelles	*Ldlr^−/−^* mice (C57Bl/6)		8.0 ± 0.5% release within 48 h	NF-κB	Reduce cytotoxicity	Anti-inflammatory	[33]
CIN	C_10_H_18_O	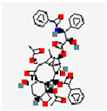	CIN@DEX_5k_-BSA/PTM/VB_12_		Macrophages	Good stability in stimulated gastrointestinal environment	PPAR-γ NF-κB	Improve CIN stability; increase oral bioavailability	Anti-inflammatory	[40]
			MM-CIN-BDS	C57BL/6 mice	AS plaque site		PPAR-γ NF-κB	Improve CIN stability	Anti-inflammatory; improves blood lipid levels	[41]
TanIIA	C_19_H_18_O_3_	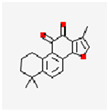	pHDL—TanIIA	ApoE^−/−^ mice	AS plaque site	Good stability under physiological conditions within 12 h		Improve TanIIA water solubility	Anti-inflammatory; regulates dyslipidemia	[45]
COL	C_22_H_25_NO_6_	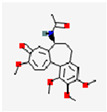	VHPK-PLGA@COL	ApoE^−/−^ mice	VCAM-1 inflammatory endothelial cells	~70.73% release at 37 °C; ~7.55% at 4 °C	NF-κB/NLRP3	Sustain drug release	Anti-inflammatory	[48]
BBR	C_20_H_18_NO_4_	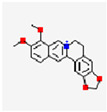	BBR NPs@Man/M2	ApoE^−/−^ mice	Inflammatory sites	~92.35% BBR release in 20% FBS within 60 h		Sustain drug release; enhance BBR bioavailability	Anti-inflammatory; promotes vascular endothelial repair	[51]
GP and EM	C_17_H_24_O_10_ C_15_H_10_O_5_	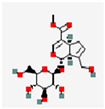	TK-MLP@ (GP + EM) NPs	ApoE^−/−^ mice	AS plaque site	~86.5% GP release and ~64.2% EM release in 1 mM H_2_O_2_ within 72 h	RONS/NF-κB/NLRP3	Prolong circulation half-life Triggers controlled drug release	Protect endothelial cells; promotes cholesterol efflux Reduces lipid accumulation	[59]
		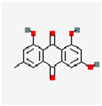								
ART and Pc	C_15_H_22_O_5_ C_30_H_26_O_13_	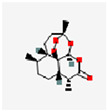	HA-M@PB@ (PC + ART) NPs	ApoE^−/−^ mice	Inflammatory macrophages	~86.5% GP release and ~64.2% EM release in 1 mM H_2_O_2_ within 72 h	RONS/NF-κB/NLRP3 AMPK/mTOR	Large drug accumulation in AS plaque	Suppress lipid influx; promote cholesterol efflux	[69]
		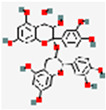								
VC	C_6_H_8_O_6_	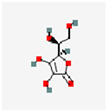	VC@cLAVs	SD rat ApoE^−/−^ mice	Foam cells	VC-LA mutual regeneration cycle		Prolong blood half-life; enhance antioxidant capacity	Scavenge ROS; inhibit cellular ox-LDL uptake	[73]
ASX	C_40_H_52_O_4_	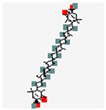	ASX-PLGA NPs	ApoE^−/−^ mice	Foam cells	58.7% release after 8 h	MAPK pathway	Improve ASX solubility; achieve local sustained release	Inhibit ferroptosis; alleviates cellular oxidative stress	[60]

**Table 2:** Key parameters of BNPs with NDDS for AS Therapy.

Classification	NDDS	Natural Drug Monomer	Materials	Preparation Methods	Particle Size	Zeta Potential	Encapsulation Efficiency (EE%)	Drug Loading (DL%)	Pharmacokinetic Properties	References
Polymer-based nanoparticles	Cel-loaded PEG-b-PP micelles (Cel-MC)	Cel	PEG PPS	Film hydration method	16.4 nm		Highest close to 100%	0.22%	Prolongs in vivo half-life	[33]
	VHPK-PLGA@COL	Col	PLGA VHPK PEG	Double emulsion method (W/O/W)	187.50 ± 1.71 nm	−33.56 ± 1.82 mV	93.32 ± 1.14%		Prolongs in vivo circulation time Stable plasma concentration within 48 h	[48]
Polymer-based nanoparticles	ASX-PLGA NPs	ASX	PLGA	Emulsification solvent evaporation	109.13 ± 0.81 nm	−27.44 ± 1.52 mV	57.00%	0.07%	Prolongs in vivo circulation time Stable plasma concentration within 48 h	[60]
Cell membrane-coated nanoparticles	MM@NPs	RSV	CLIKKPF β-CD MM	Macrophage membrane spontaneously encapsulates drugs	~231.97 nm	−27.44 ± 1.52 mV	12.80%		Prolongs in vivo half-life (3.1 h–6.3 h) Fluorescence signal intensity in target tissues increased by 2.7 times	[17]
	MM-CIN-BDS	CIN	PLGA Polyvinyl alcohol DEAE THP-1	Co-extrusion method	192.14 ± 3.39 nm	28.46 ± 0.42 mV	73.90 ± 1.51%		Increases distribution in plaque areas Reduces distribution in heart and kidneys	[41]
	BBR NPs@Man/M2	BBR	PLGA Cell membrane of M2 macrophages Man	Nanoprecipitation method Membrane fusion technology	230 nm	−26.1 ± 0.6 mV	73.90 ± 1.51%		Prolongs in vivo circulation time Enriched in the chest of AS mice after 6 h	[51]
	TK-MLP@ (GP + EM) NPs	GP and EM	The macrophage membrane (Møm) Nano-liposomes TK	Film hydration method Membrane fusion technology	184.6 nm	−46.93mV	GP 87.4% EM 62.5%		Circulation half-life t_1_/_2_ prolonged by about 77.8% Fluorescence intensity in target tissues increased to 1.91 times	[59]
	HA-M@PB@ (PC + ART) NPs	ART and Pc	Møm Red blood cell membranes HA	Membrane fusion technology	150.3 ± 2.5 nm	−7.21 ± 0.18 mV	PC: 74.21%		Prolongs in vivo circulation time by about 67.2% Reduces immune clearance	[69]
Biomolecular material nanoparticles	HASF@Cur	Cur	oHA TKL Fc	Self-assembly method	150.8 nm	−35.04 mV	51.41%	0.05%	Prolongs circulation half-life Increases accumulation in AS plaque areas	[15]
	BC@CS/cRGD NPs	BC	NH_2_NH_2_·H_2_O CS cRGD	Combining BC with CS and modifying with cRGD peptide	214.8 ± 13.4 nm	−18.7 ± 2.67 mV	81.22%		Specific accumulation in AS plaque areas	[26]
	CIN@DEX_5k_-BSA/PTM/VB_12_	CIN	DEX-BSA PTM VB_12_	Microfluidization combined with ultraviolet irradiation	100 nm	≈0 mV			Prolongs residence time in the small intestine Increases enrichment in AS plaque areas	[40]
	pHDL—TanIIA	TanIIA	DMPC Solutions of mimetic peptides	Microfluidic technology	15.5 ± 2.76 nm	≈0 mV	93.19 ± 1.14%	9.09% ± 0.01%	Prolongs in vivo circulation time Increases plaque area enrichment (28.3 times)	[45]
	VC@cLAVs	VC	VC LA 1,4,7-Triazanonane	Self-assembled vesicles	200 nm	About 10 mv		0.60%	Long in vivo half-life High in vivo exposure (AUC: 2823.9 μg·h·L^−1^)	[73]
Inorganic nanoparticles	Cur-MnO_2_/HA	Cur	HA MnC_l2_ NaOH	Ultrasonication Centrifugation Co-incubation drug loading	~230 nm	−20 mV to −30 mV, approximately −23 mV		0.54%	Significantly prolongs Cur half-life by 6 times Increases accumulation in lesion areas by 3.5 times	[16]

**Table 3:** Delivery strategies of BNPs with NDDS for AS treatment.

Targeting Strategy	Nanoparticle Name	Targeting Strategy	Target	Reference
Structural Modification	HASF@Cur Micelles	oHA modification	CD44 receptors	[15]
	Cur-MnO_2_/HA System	HA modification	CD44 receptors	[16]
	SDP-VCAM-1/Cur Particles	Conjugation of VCAM-1-targeting peptides	VCAM-1	[77]
	MP-QT-NPs	Host–guest interactions between β-CD and ADA	Macrophages	[22]
	BC@CS/cRGD NPs	cRGD peptide modification	α_v_β_3_ receptors	[26]
	LDE	Remove ApoB100	LDLR/LRP	[37]
	CIN@DEX_5k_-BSA/PTM/VB_12_	VB_12_ binding to intrinsic factor (IF)	VB_12_ receptors	[40]
	VHPK-PLGA@COL	VHPK peptide modification	VCAM-1	[48]
	MCMN-DHA-a1	Surface-modified anti-PECAM-1	Inflammatory endothelial cells	[78]
Biomimetic Targeting	MM@NPs	Macrophage membrane coating	VCAM-1	[17]
	pHDL-TanIIA and pHDL-Cur	Utilization of the inherent targeting ability of HDL	ABCA1 ABCG1 SR-B1	[45]
	MM-CIN-BDS	Monocyte membrane encapsulation	VCAM-1 ICAM-1 L-selectin	[41]
Passive Targeting	NLCE/CSNLCE	EPR effects Chitosan coating	Macrophages	[79]
	PEG-b-PPS	EPR effects Spherical shape Amphiphilic structure	Macrophages Dendritic cells	[33]
	VC@cLAVs	Passive targeting	Atherosclerotic plaques	[73]
	CDNVs	Extracellular vesicle characteristics	Endothelial cells Inflammation-related cells	[80]
Composite Targeting	BBR NPs@Man/M2	The “homing” effect of M2-type macrophage membranes Mannose-targeting peptides	Macrophages Endothelial Cells	[51]
	TK-MLP@ (GP + EM) NPs	TK-modification ROS-responsive surface linkers	Macrophages Endothelial Cells	[59]
	HA-M@PB@ (PC + ART) NPs	RBCm and Møm camouflage HA modification	CD44 receptors	[69]

**Table 4:** Potential drug candidates applicable to NDDS.

Category	Drug	Molecular Formula	Structure	Limitations	Cellular Model	Animal Model	Effects	Mechanisms and Signaling Pathways	References
Antioxidant and Anti-inflammatory	Salidroside (SAL)	C_14_H_20_O_7_	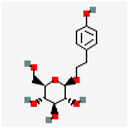	Poor targeting ability; bioavailability affected by formulation	ox-LDL-induced HUVEC injury model		Reverse ox-LDL-induced cell damage Increase antioxidant enzyme activity Improve mitochondrial dysfunction	Activate AMPK/SIRT1 pathway	[94]
					LPS and ATP-induced HUVECs model	ApoE^−/−^ mice	Reduce the area of aortic plaque Inhibit pyroptosis	Inhibit NLRP3-associated pyroptosis Suppress caspase-1 activation and IL-1β release	[95]
	Luteolin (LUT)	C_15_H_10_O_6_	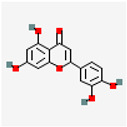	Pharmacokinetic limitations	H_2_O_2_-induced A7r5 cell model	HFD and vitamin D3-induced SD rat vascular calcification model	Improve vascular calcification Inhibit oxidative stress	Activate SIRT1 Inhibit oxidative stress Promote autophagy to alleviate vascular calcification	[96]
					ox-LDL-induced mouse primary macrophages	ApoE^−/−^ mice	Reduce plaque area Inhibit pro-inflammatory factors	Suppress STAT3 phosphorylation	[97,98]
Inhibiting Cell Migration/Proliferation	Icariin (ICA)	C_33_H_40_O_15_	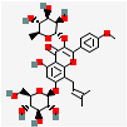	Low bioavailability	HAVSMCs	ApoE^−/−^ mice	Inhibit plaque formation Suppress HAVSMC proliferation/migration	Upregulate miR-205-5p to target ERBB4 Inhibit ERBB4/AKT signaling pathway	[99]
					ox-LDL-induced HUVECs		Inhibit ox-LDL-induced EndMT	Suppress miR-148b-3p Upregulate ELF5 Inhibit EndMT	[100]
	Evodiamine (EVO)	C_19_H_17_N_3_O	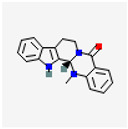	Extensive first-pass effect; hepatotoxicity	MOVAS cells	LDLR^−/−^ mouse	Reduce the area of aortic plaque Inhibit VSMC proliferation/migration Suppress inflammation and oxidative stress	Inhibit PI3K/Akt signaling pathway Reduce inflammation and oxidative stress Suppress VSMC proliferation	[101]
						PDGF-BB-induced rat VSMC model	Inhibit PDGF-BB-induced VSMC migration	Activate PPARγ expression Suppress the expression of migration-related proteins in VSMCs	[102,103]
Regulating Lipid Metabolism	Leoligin (LEO)	C_27_H_34_O_7_	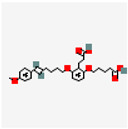	Potential endothelial damage	Human and rabbit plasma samples	CETP transgenic mice	Increase CETP activity	Bind CETP to enhance its activity Promote cholesterol transformation	[104]
					THP-1 macrophage model		Increase apoA1-mediated cholesterol efflux Upregulate ABCA1/ABCG1 without affecting SR-B1	Enhance ABCA1/ABCG1 transcription Elevate mRNA levels Promote cholesterol efflux	[105,106]
	Oridonin (ORI)	C_20_H_28_O_6_	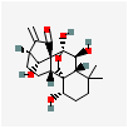	Low oral bioavailability	Raw 264.7 macrophages	ApoE^−/−^ mice	Reduce the area of aortic plaque Inhibit inflammation	Regulate LXRα-induced ABCA1 expression Promote PPARγ expression Inhibit NF-κB translocation	[107]
					Mouse peritoneal macrophages	ApoE^−/−^ mice	Reduce the area of aortic plaque Stabilize plaques Decrease lipid deposition in macrophages Suppress inflammation	Inhibit NLRP3 activation; upregulate ABCA1/ABCG1 Reduces CD36	[108,109]
